# „Die beste Alkoholprävention ist die Antiemanzipation“: Die Debatte um geschlechtsspezifischen Alkoholkonsum und die zunehmende Abhängigkeit der Frau, 1950–1990

**DOI:** 10.1007/s00048-024-00407-z

**Published:** 2024-11-19

**Authors:** Viola Balz

**Affiliations:** 1 Professur für Klinische Psychologie und Psychosoziale Beratung, Evangelische Hochschule Dresden, Dresden, Deutschland; 2 Institut für Geschichte der Medizin und Ethik in der Medizin, Charité-Universitätsmedizin Berlin, Berlin, Deutschland

**Keywords:** Frauenalkoholismus, Emanzipation, Psychologie, Soziale Arbeit, Normalität, Women’s alcoholism, Emancipation, Psychology, Social work, Normality

## Abstract

Der Beitrag widmet sich aus einer geschlechterhistorischen Perspektive der Geschichte von sowie Diskussionen über einen konstatierten Anstieg von Frauenalkoholismus in der Bundesrepublik. Seit den 1950er Jahren beklagt der psychiatrische, pädagogische und psychologische Diskurs einen zunehmenden weiblichen Alkoholkonsum und macht die Frauenemanzipation als dessen Ursache aus. Der Artikel beleuchtet die männlich dominierten Debatten über Frauenalkoholismus bis 1968 sowie anschließend einsetzende feministische Kritiken. Er analysiert die in den Diskussionen über „die trinkende Frau“ zum Ausdruck kommenden Veränderungen der gesellschaftlichen Rolle von Frauen sowie zeitgleich sich formierende wissenschaftlich-patriarchale Gegenbewegungen. Dabei zeigt der Beitrag zum einen, wie ein klassischer Suchtbegriff aufgrund des Scheiterns medizinischer Behandlungsversuche erodiert und durch neues psychosoziales Erklärungswissen ersetzt wird. Zum anderen verdeutlicht er, wie die Frauenselbsthilfe sich dieses Wissen aneignet und neu interpretiert.

## Abstract (en)

From a gender-historical perspective, this article deals with the history of and discussions around an observed increase in female alcoholism. Since the 1950s, psychiatric, pedagogical and psychological discourses have lamented the increasing consumption of alcohol by women, and identified women’s emancipation as its cause. The article examines the male-dominated debates on female alcoholism up to 1968 and the emerging feminist counter-movement that followed. It analyzes the shifts in the social role of women as expressed in the discussions about ‘the drinking woman’ as well as simultaneous scientific-patriarchal counter-movements. On the one hand, the article shows how a classic concept of addiction is eroding due to the failure of medical treatment attempts and is being replaced by new psychosocial explanatory knowledge. On the other hand, it illustrates how women’s self-help is appropriating and reinterpreting this knowledge.

„Im 12. Und 13. Jahrhundert haben sich Männer darüber gestritten, ob die Frau eine Seele habe oder nicht und sind schließlich zum Ergebnis gekommen, sie habe keine. Vielleicht mögen die Herren Wissenschaftler auf dem Hintergrund der jetzigen Erkenntnisse ihre Aussagen etwas relativieren, um nicht irgendwann wie Freud nach 30 Jahren Arbeit feststellen zu müssen, daß er nichts, aber auch wirklich nichts über das Wesen der Frau wisse.“^1^

Mit diesen Worten fasst eine Teilnehmerin die Ergebnisse einer 1980 stattfindenden Tagung der „Deutschen Hauptstelle gegen Suchtgefahren“ (DHS) zum Thema Frauen und Sucht zusammen. Die Konferenz zeichnet den zunehmenden Alkoholkonsum von Frauen als Folge der Emanzipationsbestrebungen nach und weist auf die damit einhergehenden Belastungen für Frauen hin. Die Beteiligten knüpfen dabei an historische Diskussionen um Alkoholkonsum und Geschlecht an und führen diese fort, indem sie den Alkoholkonsum von Frauen als „unweiblich“ und abweichend markieren. Generell ist im 20. Jahrhundert die Debatte über Suchterkrankungen im psychiatrischen Diskurs stark mit Geschlecht und Geschlechterstereotypen verbunden: Aufgrund der hohen Zahl der Betroffenen wird Alkoholismus als Krankheit des Mannes bezeichnet (Schwamm 2018; Pfütsch 2015). Der abhängige Konsum von Tranquilizern gilt hingegen wegen der hohen Zahl an Konsumentinnen als weiblich (Tone 2009). Aufgrund der stark ungleichen Geschlechterverteilung lassen sich Geschlechterstereotype am Beispiel des Alkoholismus besonders gut aufzeigen.

Anders als die bisherige Forschung konzentriere ich mich in diesem Artikel nicht auf den männlichen Alkoholismus in der Bundesrepublik, obwohl dieser auch in meinem Untersuchungszeitraum von 1950 bis 1990 statistisch dominant bleibt. Vielmehr möchte ich mich aus geschlechterhistorischer Perspektive insbesondere der Geschichte des Frauenalkoholismus und den Diskussionen um dessen konstatierte Zunahme widmen. Wie ich am Beispiel der Entstehung neuer wissenschaftlicher und gesellschaftlicher Debatten über weiblichen Alkoholismus – und der Versuche, diesen vorzubeugen – aufzeige, wird die Assoziation von Alkoholismus und Männlichkeit seit den 1950er Jahren brüchig. Mit dem zunehmenden gesellschaftlichen Wohlstand in den 1950er und 1960er Jahren wird im psychiatrischen, pädagogischen und psychologischen Diskurs ein steigender Alkoholkonsum beklagt, der der Frauenemanzipation angelastet wird. Mit dem wachsenden Einfluss sozialer Bewegungen weiten sich die Diskussionen über problematisches Trinken seit den 1960er Jahren stark aus.

Die gesellschaftlichen Debatten über die „trinkende Frau“ sind auch deshalb besonders interessant, weil sich darin Verschiebungen der gesellschaftlichen Rolle von Frauen sowie der entsprechenden wissenschaftlich-patriarchalen Gegenbewegungen widerspiegeln. So gelten Frauen etwa in der Gesundheitsaufklärung lange Zeit als Vorreiterinnen gesundheitsbewussten Verhaltens. An ihnen sollen die Defizite männlichen Gesundheitsverhaltens gemessen werden; Frauen sollen Männern als Vorbild dienen, wie Pierre Pfütsch (2015: 177f.) in einer Analyse der Programme der Bundeszentrale für gesundheitliche Aufklärung (BZgA) aufzeigt. Während zahlreiche historische Arbeiten zu Alkoholismus als männliche Krankheit vorliegen, ist die Beschäftigung mit Frauenalkoholismus ein blinder Fleck in der historischen Forschung (Schwamm 2018; Pfütsch 2015; Linnek 2016). Schon Elke Hauschildt (1995: 24) stellt für die bundesrepublikanische Nachkriegszeit fest, dass „der Trinker“ in zeitgenössischen Veröffentlichungen sowie in der beschriebenen Praxis per definitionem männlich ist. Weiblicher Alkoholismus gelte als so außergewöhnlich, dass er in der Forschung weitgehend ignoriert worden sei (ebd.).

In diesem Beitrag gehe ich *erstens *der Frage nach, wie sich im Zuge der Diskussionen um die Geschlechterordnung in der Nachkriegszeit die Differenzkonstruktionen von männlich/weiblich sowie normal/verrückt beziehungsweise normalem/pathologischem Alkoholkonsum verschieben. *Zweitens* frage ich, inwiefern im Untersuchungszeitraum eine psychologische und pädagogische Neuausrichtung des Wissens über normales oder pathologisches Alkoholtrinken erkennbar ist. Dafür zeichne ich anhand psychiatrischer, psychologischer und pädagogischer Publikationen die Debatte über ein angemessenes Trinkverhalten von Frauen nach. Meine Analyse konzentriert sich dabei auf die Bundesrepublik, wird aber ergänzt durch einzelne für den bundesdeutschen Diskurs zentrale Fachbeiträge aus Österreich und der Schweiz.^2^ Als Quellen dienen dabei einschlägige Diskussionen in den beiden zentralen sozialpädagogisch-fürsorgerischen Zeitschriften *Soziale Arbeit* und *Blätter zur Wohlfahrtspflege*, in der für den Suchtbereich maßgeblichen interdisziplinären Zeitschrift *Suchtgefahren: Beiträge aus Fürsorge und Forschung* (herausgegeben von der DHS) sowie einzelne Artikel aus psychiatrischen Zeitschriften.^3^ Da die DHS die zentrale Dachorganisation aller Einrichtungen und Verbände ist, die sich seit Mitte der 1950er Jahre der Fürsorge für Alkoholgefährdete und Alkoholkranke widmet, werte ich auch die einschlägigen Kongressbeiträge dieser Organisation aus. Um die feministischen Deutungen dieses Diskurses zumindest in Ansätzen zu erfassen, skizziere ich die Rezeption dieser Forschung in den populären und feministischen Medien.^4^ Ergänzend ziehe ich Archivalien der BZgA heran. Wie ich zeigen werde, finden die Diskussionen zumindest zu Beginn des Untersuchungszeitraums vor allem in den sozialpädagogisch-fürsorgerischen Zeitschriften statt. Für die Bundesrepublik lässt sich somit schon vor 1968 eine Öffnung des Feldes der Suchthilfe auf Soziale Arbeit und Theologie beobachten, anders als zum Beispiel in der Schweiz (Herzig 2022: 75–79).

Die analysierten Beiträge knüpfen an medizinisch-psychiatrische Diskussionen aus der Zeit um 1900 an. Bereits der Sozialhygieniker Alfred Grotjahn (1898) behauptete Ende des 19. Jahrhunderts, dass weiblicher Alkoholismus mit einem endogenen Faktor angeborener geistiger Minderwertigkeit zu erklären sei. Der Psychiater Emil Kraepelin erklärte noch Ende der 1920er Jahre, die Frau wüchse in geschützteren Verhältnissen auf, daher sei weibliche Trunksucht als anlagebedingt und eben nicht als Teil zermürbender Umweltanforderungen zu begreifen (1927). Den sogenannten Elendsalkoholismus erklärten Forscher:innen lange Zeit mit den elenden und harten sozialen Bedingungen (Engels 1962 [1845]), schrieben ihn aber vor allem Arbeitern und weniger Arbeiterinnen zu. Frauen kam dabei eher die Rolle von Gesundheitshüterinnen zu. Angelehnt an entsprechende Entwicklungen in den USA im 19. Jahrhundert wurde auch in Deutschland in den 1930er und 1940er Jahren eine als Temperenzbewegung bezeichnete Aufforderung zur Abstinenz stark, bei der Frauen ihre Ehemänner vor den Gefahren von Alkohol und Tabak beschützen wollten und diese Prävention stark mit erbbiologischen Fragen und einer Degenerationslehre verbanden (Linhardt 1941). Diese Bewegung ist vor dem Hintergrund zu betrachten, dass „erbbiologisch minderwertige Trinker“ seit Mitte der 1930er Jahre als unheilbar galten und seit 1940 der Gefahr einer Vernichtung durch das NS-Regime ausgesetzt waren. „Erbbiologisch nicht minderwertige Trinker“ hingegen wurden als heilbar bezeichnet; auf Letztere konzentrieren sich auch die fürsorgerischen Maßnahmen (Hauschildt 1995: 116–118). Riskanter Substanzkonsum, insbesondere von Alkohol, galt an sich bereits als eine Praktik der Männlichkeit (Schwamm 2018: 75). Dies änderte sich jedoch ab den 1950er Jahren.

Der Beitrag beleuchtet zunächst die durch Männer dominierten Debatten über eine konstatierte Zunahme von Frauenalkoholismus in den 1950er und 1960er Jahren. Anschließend skizziert er die neuere Rechtssprechung zum Alkoholismus, die eine Neuausrichtung der Versorgung von Alkoholiker:innen ermöglicht. Wie ich nachzeichne, wird der Frauenalkoholismus in den 1970er Jahren zunehmend zum wissenschaftlichen und medialen Phänomen. Zeitgleich widmen sich aber auch Feminist:innen verstärkt dem Thema des weiblichen Alkoholkonsums. Beide Debatten kulminieren 1980 in einer kontroversen Diskussion über weiblichen Alkoholismus und dessen (männliche) Erklärungsversuche. Dabei gelingt es Feminist:innen insbesondere in den 1980er Jahren, ein Gegenwissen über Alkoholismus zu etablieren.^5^

## Von Wohlstandsalkoholismus und Männern in Not: Die 1950er und 1960er Jahre

Bereits 1950 setzt sich der Ausschuss für Alkoholismus der Weltgesundheitsorganisation für eine Verbesserung der Ausbildung von Gesundheitsfürsorger:innen ein und fordert, dass diese klinisch an Alkoholikerabteilungen des Krankenhauses ausgebildet werden sollten. Als Minimalausstattung jeder Alkoholikerambulanz solle mindestens ein/e Gesundheitsfürsorger:in eine/n Mediziner:in unterstützen (Habernoll 1956: 14, 29). Wohl auch vor diesem Hintergrund gibt die Landesstelle Berlin gegen Suchtgefahren 1954 eine Einführung für Fürsorger:innen und Laienhelfer:innen heraus, die sich mit der Bekämpfung der Trunksucht beschäftigt und der Fortbildung dient. Wie es in der Dokumentation heißt, sei es von zentraler Bedeutung, die Alkoholkrankenfürsorge zu intensivieren, da die Zahl der in den Fürsorgestellen zu betreuenden Trinker von Monat zu Monat wachse (Meyer 1954: 1, 7). Für den Konsum von Alkohol und damit auch als Zielgruppe für die Alkoholikerfürsorge käme jedoch, wie der Sozialhygieniker Erich Schröder (1893–1968) in dem Heft ausführt, nur eine Bevölkerungsgruppe infrage, „die man rechnerisch auf die 15- bis 70-jährigen Männer beschränken kann“ (Schröder 1954: 12). Eine ausführliche Darstellung der Arbeit mit Alkoholkranken aus der Sicht eines Oberfürsorgers betont die menschlichen und materiellen Hilfen, die den Betroffenen ein Leben in Würde ermöglichen sollten. Dabei sei die Lebensweise des Trinkers zu akzeptieren, solange er nicht andere gefährde (Winkel 1954: 84–86). Tatsächlich wurde erst im Lauf der 1950er Jahre wieder vermehrt Alkohol getrunken. Wie der Jenaer Psychiater Hugo von Keyserlingk mit Bezug auf beide deutschen Staaten ausführt, sei der Alkoholkonsum während des Krieges und in der direkten Nachkriegszeit durch Beschaffungsschwierigkeiten und später durch den hohen Preis sichtlich zurückgegangen und erst seit Anfang der 1950er Jahre erneut gestiegen (von Keyserlingk 1959: 1–3).

Diesen Anstieg der Alkoholkrankheiten mit Beginn des wirtschaftlichen Aufschwungs thematisieren Anfang der 1950er Jahre auch Zeitschriften der öffentlichen Fürsorge. Erstmals adressieren sie auch direkt Frauenalkoholismus: „Der Alkoholverbrauch, früher ausgesprochene ‚Männerangelegenheit‘, ist in den letzten Jahren mehr und mehr aus der ‚Öffentlichkeit der Gaststätte‘ in den häuslichen Kreis der Familie abgewandert“ (Anonymus 1954: 432). Diese Privatisierung des Alkoholkonsums mache es auch Frauen und Jugendlichen leichter, mehr zu konsumieren. Im Jahr 1953 hätten die Bundesbürger:innen fast sechs Millionen D‑Mark für Alkohol ausgegeben – ungefähr ein Viertel dessen, was sie insgesamt für Nahrungsmittel aufwenden (ebd.). Der Pro-Kopf-Verbrauch von reinem Alkohol lag 1950 bei etwa drei Litern pro Person (Schott & Tölle 2006: 348). Dies führe, wie die Zeitschrift *Suchtgefahren* konstatiert, zu einem veränderten Trinkverhalten von Frauen:

„Die soziale Stellung der Frau hat sich in den letzten Jahrzehnten ganz wesentlich geändert. Die sozialen Umwälzungen brachten es mit sich, daß die Frau heute fast alle Berufe, die früher den Männern vorbehalten waren, ausübt und daher auch die Freiheiten und Gewohnheiten des Mannes für sich in Anspruch nimmt. Das macht sich besonders bemerkbar in der Großzügigkeit sexueller Auffassungen […] und nicht zuletzt auch durch die Nachahmung „männlicher“ Trinkmuster. So kam es in so gut wie allen zivilisierten Ländern zu einer Häufigkeitssteigerung des Alkoholismus der Frau“ (Gabriel 1957: 4).

Der Artikel beklagt, dass bereits zehn Prozent der wegen Alkoholsucht Behandlungsbedürftigen weiblich seien. In Großstädten liege ihr Anteil noch weit höher, zudem sinke das Alter des ersten Alkoholkonsums bei Frauen stetig. Zwei Drittel der Trinkerinnen seien ledig, geschieden oder verwitwet, nur ein Drittel lebe in einer Ehe. Dagegen seien männliche Trinker deutlich häufiger verheiratet (ebd.).

Grundsätzlich wird der Alkoholismus seit dem 19. Jahrhundert als eine Krankheit des Willens beschrieben, dem daher auch nur mit einer Stärkung der Willenskraft sowie mit sittlich-moralischer Erziehung begegnet werden könne (Valverde 1998). Diese Zuordnung rückt die Trinker:innen in die Nähe von Asozialen und Kriminellen, die sich gewissermaßen schuldhaft selbst krank gemacht hätten. Zu Beginn des Untersuchungszeitraums sind Alkoholiker:innen vor allem im Blickfeld von Polizei, Fürsorge und Medizin. Sie sind eingebunden in ein enges System aus Zwang und Kontrolle (Hauschildt 1995: 233). Bis Anfang der 1950er Jahre dominieren Versuche der Psychiatrie, die Trunksucht als somatische Krankheit zu etablieren – mit entsprechenden somatisch-erbbiologischen Deutungsversuchen. Dagegen sprechen Psychiater bereits seit Ende der 1950er Jahre von Alkoholismus als einer gesellschaftlichen Erscheinung. Auch die in den 1950er Jahren eingeführte medikamentöse Antabus-Kur bringt nicht die erwünschten Erfolge. Die verabreichten Tabletten dämmen das Verlangen nach Alkohol nur kurzfristig ein und ersetzen keine Entziehungskur, die wiederum auf die nachhaltige Stärkung der Willenskraft der Einzelnen setzt (ebd.: 222f.). Aufgrund der historisch engen Verknüpfung der Sozialen Arbeit mit der Diakonie dominieren gerade in den 1950er und 1960er Jahren Theolog:innen und christlich-moralische Fragen die Fürsorgearbeit. Gleichzeitig beginnt sich die Fürsorge im Gesundheitswesen stärker zu etablieren. Dies zeigt sich nicht nur in der Gründung zur Vereinigung eines Fürsorgedienstes im Krankenhaus, sondern auch in der Etablierung einer einjährigen Fortbildung zur psychiatrischen Fachfürsorger:in im Jahr 1957, die in die Methode der Einzelfallarbeit (case work) einführen und neben sozial- und tiefenpsychologischem Wissen auch psychiatrische Grundkenntnisse vermitteln soll (Anonymus 1959). Mit der Einführung des Bundessozialhilfegesetzes im Jahr 1961 wechselt die offizielle Berufsbezeichnung von „Wohlfahrtspflege“ zu „Soziale Arbeit“; eine dreijährige Ausbildung und ein anschließendes Praktikum sind nun vorgesehen (Brückner 2021: 21).

Gerade im Bereich der Alkoholfürsorge bleiben jedoch Versorgungsengpässe bestehen. Da es der staatlichen Gesundheitsfürsorge in den Nachkriegsjahren nicht gelingt, die ambulante Nachsorge bei der Alkoholismusbehandlung an das höhere Vorkriegsniveau anzupassen, nehmen die Verantwortlichen die ehrenamtlichen Bemühungen christlicher Abstinzenzlerverbände dankbar an. Bereits seit Ende des 19. Jahrhunderts ist die Kirche mit dem Verein „Das Blaue Kreuz“ in der Arbeit mit Alkoholiker:innen aktiv. Der Verein ist strikt abstinenzorientiert und setzt auf die Bekehrung der „Trunksüchtigen“ durch Gebet und Glauben. 1962 konstatiert das Blaue Kreuz, dass fast jede fünfte trunksüchtige Person weiblich ist (Klement 2010: 170–175). Die christliche Orientierung und der Fokus auf vermeintliche moralische Verfehlungen kennzeichnet in den 1950er und 1960er Jahren auch die Diskussion in den *Blättern der Wohlfahrtspflege*. Diese widmen dem Thema Alkoholismus 1958 sogar einen eigenen Schwerpunkt. Der Stuttgarter Medizinaldirektor Richard Cario (1958) stellt darin fest, dass den Süchtigen in der modernen Welt zuvor Bindung und Halt verloren gegangen seien. Auf der Flucht vor sich selbst gerieten die Betroffenen dann in die Sucht. Auch ein Pastor konstatiert die Sucht als Nebenerscheinung des modernen, gestressten Lebens, das durch ein Zuviel an Arbeit gekennzeichnet sei. Dem könne nur mit vollständiger Abstinenz der Betroffenen sowie mit Liebe im Raum kirchlicher Diakonie begegnet werden (Müller 1958). Der Stuttgarter Psychiatrieprofessor (Vorname unbekannt) Haug (1958) wiederum erklärt die Sucht primär mit minderwertigen Erbanlagen, wendet allerdings ein, dass man das Trinkverhalten auch durch eine tiefenpsychologische Analyse der Persönlichkeitsstruktur beeinflussen könne. Der Freiburger Caritas-Direktor Walter Baumeister postuliert, man könne den Alkoholismus niemals heilen, sondern lediglich die Süchtigen umerziehen, soweit sie objektiv erziehungsfähig und subjektiv erziehungsbereit seien (Baumeister 1958). Eine Fürsorgerin [Name unbekannt] weiß aus ihrer praktischen Arbeit zu berichten, dass die gewünschte Erziehungsfähigkeit in der Nachkriegszeit stark zurückgegangen sei. Bodenständige, ansprechbare und für religiöse Fragen offene Trinker treffe man in der praktischen Arbeit kaum noch an. Zudem hätten Trinkerinnen früher eine geringere Rolle gespielt. Die Fürsorgerin führt aus:

„Häufig waren es Frauen, die als Bardamen oder Dirnen trunksüchtig wurden, oder die infolge einer ausgesprochenen Psychopathie auf Alkohol abnorm reagierten. Heute haben wir es in unserer Arbeit mit etwa 15 % Frauen zu tun, und zwar mit Frauen, die vorwiegend aus geordneten Verhältnissen stammen und überwiegend bürgerlichen Berufen nachgehen“ (Anonymus 1958: 65).

In dieser Feststellung deutet sich bereits eine Erosion der Grenzziehung zwischen abstinenten bürgerlichen und „gefallenen“ trinkenden Frauen an. Der Alkoholkonsum scheint nun auch bürgerliche Frauen erreicht zu haben. Eine große Gefahr ist laut dem Artikel die Verlagerung des Alkoholkonsums in die eigene Wohnung. Sogar in „einfachen“ Kreisen hätte sich der Trend zur Hausbar etabliert. Ehefrauen könnten zu Hause nicht nur ungehinderter trinken, sondern auch nicht mehr so gut ihrer Aufgabe nachkommen, den Alkoholkonsum ihrer Ehemänner zu unterbinden. Gleichzeitig seien die Frauen und ihre Kinder den Gewaltexzessen ihres Mannes hier schutzloser ausgesetzt (ebd.).

Kontrovers diskutiert wird die Frage, ob die zunehmende Berufstätigkeit von Frauen die Zahl der Trinker:innen nach oben getrieben habe. Ein von Hans von der Heydt in der Zeitschrift *Soziale Arbeit* veröffentlichter Artikel (1962) konstatiert, dass unter den Alkoholikerinnen ebenso arbeitende Unverheiratete wie nicht berufstätige Ehefrauen und Mütter seien. Der Alkoholismus ziehe sich inzwischen durch alle Schichten und treffe keinesfalls nur einsame Frauen und Prostituierte. Um zu verstehen, warum Frauen trinken, müsse man daher die sich wandelnde Stellung von Frauen in der Gesellschaft näher betrachten. Der Autor macht insbesondere die Gleichberechtigung für den zunehmenden Alkoholismus verantwortlich: Diese habe es erforderlich gemacht, dass sensible und leicht verletzbare Frauen in männlichen Wirkungskreisen arbeiten müssten. Zuvor hätten Frauen im geschützten Familienkreis agiert und seien deshalb weniger anfällig für Alkoholismus gewesen. Von der Heydt schließt damit direkt an die These Kraepelins an, wonach Frauen durch ihre Tätigkeit in der privaten Sphäre des Haushalts generell geschützt seien (ebd.). Über die soziale Bedingtheit des Alkoholismus berichtet auch die Zeitschrift *Suchtgefahren*. Den steigenden Suchtmittelkonsum betrachtet sie jedoch eher als Folge von zunehmendem Wohlstand und mehr Freizeit. Nicht gefestigte Menschen wüssten damit nichts anzufangen, was sie zu immer mehr Konsum verleite – auf Kosten moralischer Werte (Gabriel 1963). Einige Jahre später führt Ilse Szagunn (1965) aus, dass die Beschäftigung mit dem Thema Alkohol gerade wegen des zunehmenden Alkoholkonsums von Frauen aus sozialmedizinischer und ökonomischer Sicht drängender werde, da dieser zu einer steigenden Zerrüttung von Familien führe. Schon in diesen frühen Diskussionen wird beklagt, dass Frauen durch ihren Alkoholkonsum aus ihrer Rolle als schützende Ehefrauen und Familienhüterinnen auszubrechen drohen.

Das verweist auf einen weiteren Diskurs, der in den 1950er Jahren bereits seine Anfänge nahm, sich jedoch erst in den 1960er Jahren voll entfaltete und sich mit der vermeintlichen Verantwortlichkeit der Frau für das Trinkverhalten ihres Ehemannes befasste. Kampagnen zur Senkung des Alkoholkonsums richteten sich dementsprechend an Frauen nicht nur als Konsumentinnen, sondern auch als für das Trinkverhalten ihres Partners Verantwortliche. Beispielhaft sei hier das Buch *Männer in Not* (Schmidt 1962) des christlich-protestantischen Blaukreuzverlags genannt, dem Verlag des gleichnamigen Vereins. Es erzählt zahlreiche Geschichten von schweren Trinkern, die durch Gebete und tugendhaftes Verhalten beeinflusst und wieder auf den Pfad des rechten Lebens zurückgeführt werden konnten. In dem Buch heißt es, den zu Rettenden sei mit Liebe und Zartheit zu begegnen. Frauen werden dazu angehalten, ihre trinkenden Ehemänner nicht zum Zorn zu reizen. Ihnen wird sogar geraten, notfalls handgreiflich zu werden, wenn es erforderlich sei, um die Männer vom Alkohol abzubringen (ebd.: 57). Dass die Frau für ein angemessenes Trinkverhalten des Mannes sorgen solle, behaupten auch andere Publikationen aus dem Fürsorgebereich – etwa die Zeitschrift *Soziale Arbeit*. Diese macht eine schlechte Ehe und eine mangelhafte Haushaltsführung für die Alkoholsucht des Mannes verantwortlich (Deutsch 1960). Dass Frauen ihre fürsorglich-unterstützende Hausfrauenposition verlassen und stattdessen zu ihrem eigenen Vergnügen Alkohol konsumieren, wird in den 1950er und 1960er Jahren als Anzeichen einer zusammenbrechenden Gesellschaftsordnung beklagt, die durch die zunehmende Freizeit, den Wohlstand und den Autoritätsverlust von Institutionen wie Kirche und Staat befördert werde (Gabriel 1963: 19f.).

Dominierten in den 1950er und 1960er Jahren noch solche moralischen Fragen – mit einer entsprechenden Präsenz von Theolog:innen – setzt ab Mitte der 1960er Jahre mit dem konstatierten Autoritätsverlust der Kirchen eine erste Verrechtlichung der Thematik ein. Unter anderem affirmiert die Rechtssprechung die Behauptung einer Zerrüttung von Familien durch den Alkoholkonsum der Frau. Im Oktober 1964 urteilt der Bundesgerichtshof über einen Fall des schuldhaften Scheiterns einer Ehe wegen der Trunksucht der Ehefrau. Diese gibt an, dass sie die ihr zur Last gelegten, im betrunkenen Zustand begangenen Eheverfehlungen aufgrund ihrer Alkoholsucht nicht mehr habe steuern können. Das Gericht entscheidet letztinstanzlich, dass von einem schuldhaften Verhalten infolge einer (psychopathischen) Willensschwäche auszugehen sei. Die Verurteilte hätte ihrem Drang zum Alkoholkonsum unter Aufbringung aller Willenskraft entgegentreten müssen. In der Konsequenz wird die Frau schuldhaft geschieden.^6^ Bemerkenswert ist, dass ein solches Urteil erst gefällt wird, als eine Frau dem Rausch verfällt. Die ehelichen Verfehlungen von Alkoholikern hatten vorher zu keiner vergleichbaren Rechtssprechung geführt. Zugleich reiht sich das Urteil zumindest dahingehend in die zeitgenössische Rechtssprechung ein, als es Alkoholismus als reine Willensschwäche begreift und so die Betroffenen vollumfänglich für ihre Verfehlungen verantwortlich macht. Das Bundessozialgericht urteilt etwa Anfang der 1960er Jahre, dass einem mehrfach rückfällig gewordenen (männlichen) Arbeitnehmer, der der Trunksucht verfallen ist, die Rente versagt werden kann. Erst 1968 setzt sich die Rechtsauffassung durch, dass Trunksucht als Krankheit im Sinne der gesetzlichen Krankenversicherung zu betrachten sei, die es Trinker:innen gerade nicht erlaube, mit eigener Willenskraft vom Trinken loszukommen. Dies und der Verlust der Selbstkontrolle seien Grundbedingungen für die Sucht und machten sie zu einer psychischen Krankheit.^7^ Diese Pathologisierung des Alkoholkonsums sorgt nun erstmals dafür, dass die Betroffenen das Stigma der moralischen Verfehlung loswerden.

Gleichzeitig fügt sich der Alkoholismus nicht bruchlos in eine Pathologisierungsgeschichte ein, durch die er als psychische Krankheit eingestuft wird. Wie Mariana Valverde (1998: 11) in ihrer historischen Studie zu Alkoholismus in den USA herausarbeitet, wird die Trunksucht schon im Goldenen Zeitalter der Psychiatrie – also in den 1950er Jahren – als ungeeignet für die psychiatrische Behandlung angesehen und daher an die Soziale Arbeit delegiert. In der Bundesrepublik setzt diese Sozialtherapeutisierung zögerlich ein. Doch auch hier lässt sich eine Verschiebung eines auf Zwang ausgerichteten Systems zu einer stärker therapeutisch orientierten Herangehensweise beobachten. Dazu gehört die Einführung der sogenannten therapeutischen Kette – einer Behandlungsabfolge von Suchtberatung, stationärer Entgiftung, psychosozialer Entwöhnung und Selbsthilfegruppen. Mit den Anomymen Alkoholikern sind seit Ende der 1950er Jahre auch in der Bundesrepublik konfessionslose Selbsthilfegruppen aktiv (Schott & Tölle 2006: 344, 348). Ungeklärt ist allerdings, ob der zeitgleich konstatierte Anstieg der Behandlungszahlen allein durch diese Therapeutisierung und Pathologisierung des Trinkens erklärt werden kann. Vonseiten der Sozialen Arbeit wird beklagt, dass durch die sprunghafte Zunahme des Alkoholismus die Betreuungskapazitäten völlig überlastet seien. So müsse sich die Zahl der Fürsorger:innen verdreifachen, um den gestiegenen Bedarf auch nur annähernd decken zu können (Anonymus 1967). Die von der DHS erhobenen Zahlen zeigen einen Anstieg des Pro-Kopf-Verbrauchs auf über 14 Liter reinen Alkohol 1970 (DHS o.J.). Damit hat sich der Alkoholkonsum seit den 1950er Jahren fast verfünffacht. Der Bericht einer Psychiatrie-Enquete des Deutschen Bundestags (1975) über die Lage der Psychiatrie in der Bundesrepublik dokumentiert einen starken Zuwachs der Aufnahmezahlen von Alkoholkranken in Rheinischen Krankenhäusern von 1960 bis 1970 von 513 auf 4.535. Anfang der 1970er Jahre machten Alkoholkranke und Suchtabhängige bereits 30 Prozent der Aufnahmen in psychiatrischen Kliniken aus (ebd.). Die Zahl der im Krankenhaus behandelten Alkoholdelirien steigt innerhalb weniger Jahre um 700 Prozent. Besonders gefährdet sind nach Angaben der Enquete Frauen und Jugendliche (ebd.). Mit der Einführung des Bundessozialhilfegesetzes (BSHG) 1961 kommt es erstmals zu einer Modernisierung sozialstaatlicher Hilfen. Die neu eingeführten „Hilfen in besonderen Lebenslagen“ (§ 72 BSHG) und insbesondere die Gefährdetenfürsorge eröffnen neue Perspektiven für Alkoholiker:innen. Gleichzeitig setzen Anfang der 1960er Jahre mit der Einführung von Gruppentherapien auch erste Versuche der Psycho- und Sozialtherapeutisierung von Alkoholismus ein (Hauschildt 1995: 228). Damit reagiert das Versorgungssystem auf jene Stimmen aus Medizin und Fürsorge, die den zunehmenden Alkoholkonsum und Alkoholismus bereits seit den 1950er Jahren als soziales Problem bezeichnen und entsprechende Hilfen fordern. Zugleich markiert dies eine Wende hin zu einer stärkeren Öffnung des Feldes für (sozial-)pädagogische und psychologische Fachkräfte. Um 1970 wird die Soziale Arbeit durch die Errichtung von Fachhochschulen akademisiert, zahlreiche neue fachwissenschaftliche Zeitschriften entstehen*. *Angesichts geringer finanzieller staatlicher Mittel sieht sich die Profession zunehmendem Veränderungsdruck ausgesetzt (Elberfeld 2020: 276f.). Sozialarbeiter:innen reagieren darauf zum Teil mit Versuchen, ihre Klient:innen zu therapieren. Diese Bemühungen erreichen aber kaum marginalisierte oder wenig gebildete Gefährdete, die häufig von der Alkoholikerfürsorge betreut werden. Daher werden für diese Gruppe eigene Beratungs- und Betreuungsangebote entwickelt, etwa die „Unterschichtenberatung“ (ebd.: 258–269). Erst Ende der 1970er Jahre setzten sich durch entsprechende Empfehlungen der gesetzlichen Kranken- und Rentenversicherung erste Formen der Suchttherapie – die sogenannte Entwöhnungsbehandlung – durch (Bühringer 2019).

Die Änderungen im Versorgungssystem sorgen auch dafür, dass trinkende Frauen nun als gesamtgesellschaftliches Phänomen wahrgenommen werden, das nicht mehr nur Zeitschriften der Fürsorge, sondern vermehrt auch populäre Medien diskutieren. Unter dem Titel „Arme Kreaturen, die niemand will“ beschreibt die Journalistin Angelika Grunenberg die Gefahren des Alkoholismus für Alleinstehende. Zu den Folgen führt sie aus:

„Alkoholsüchtige Frauen sind heute ein besonderes Problem. Sie sind schwieriger zu erreichen, schwieriger zu heilen und schwieriger zu resozialisieren als alkoholsüchtige Männer. Fachleute sehen den Grund hierfür in der sozialen Stellung der Frau, die – wie sie auch immer aussieht – umstritten ist. Da gibt es drei spezifische Gruppen von Alkoholikerinnen: die sogenannten Wermuthschwestern, zum großen Teil Prostituierte; die Nur-Hausfrauen und die berufstätigen Frauen. Und es gibt vor allem einen Umstand, den Ärzte, Psychologen und Soziologen für den zunehmenden Alkoholismus unter Frauen verantwortlich machten: die Emanzipation.“^8^

**Abb. 1:**
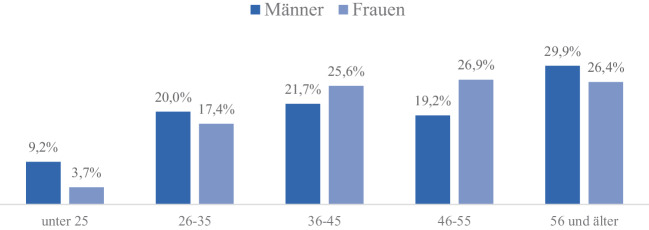
Alter der stationär behandelten alkoholsüchtigen Männer und Frauen. Daten nach Holzgreve, W. (1970), hier S. 13

Im Zuge der Sozialen Bewegungen nach 1968 macht Grunenberg auf die Kehrseite jener Emanzipationsbestrebungen aufmerksam, die Frauen aus einer untergeordneten Lage befreien und unabhängig machen sollen. Vielmehr seien die Frauen nun alkoholabhängig geworden. Es ist bemerkenswert, dass sich auch die DHS 1969 zeitgleich mit den Emanzipationsforderungen der Neuen Sozialen Bewegungen erstmalig mit dem Thema Abhängigkeit von Frauen beschäftigt. Ihre Konferenz mit dem Titel „Frauenalkoholismus – seine Ursachen, Auswirkungen und Behandlungsmöglichkeiten“ widmet sich der weltweiten Zunahme von Alkoholsucht bei Frauen. Wie der Konferenzbericht herausarbeitet, fallen dabei mehrere Tatsachen besonders auf: Frauen würden vermehrt zu Spirituosen greifen, tränken fast immer heimlich und alleine in den eigenen vier Wänden und eine Behandlung dieser Frauen sei ungemein schwierig, da sie innerhalb ihres Haushalts erst in einem viel späteren Stadium des Alkoholismus auffällig würden und entsprechende Behandlung aufsuchten. Zudem sei ein sprunghafter Anstieg des Konsums bei Frauen ab Ende 40 zu verzeichnen. Oft handle es sich bei den Alkoholikerinnen um alleinstehende Frauen; nur ein Drittel sei verheiratet. Gerade bei Letzteren steige der Konsum aber mit wachsendem Einkommen des Ehemannes an (Holzgreve 1969). Auf Grundlage der Dokumentationen der an die DHS angeschlossenen Heilstätten wertet die Organisation Daten aus den Jahren 1968 und 1969 aus, die den hohen Anteil von Hausfrauen und Frauen über 40 in der Behandlung der alkoholabhängigen Frauen belegen sollen, wie die Abb. 1 und 2 belegen.

**Abb. 2:**
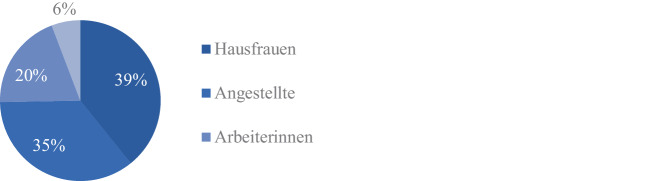
Alkoholsüchtige Frauen, die 1968/69 in Heilstätten behandelt wurden. Anteil nach Beruf, in Prozent. Daten nach Holzgreve, W. (1970), hier S. 13

Nicht nur die nun erhobenen Zahlen, sondern auch die anderen Beiträge auf der Konferenz zeigen, dass Ende der 1960er Jahre der Diskurs über trinkende Frauen zwar weiterhin von moralischen Untertönen begleitet ist, sich aber insgesamt stärker verwissenschaftlicht.

## Zwischen Wissenschaft und Öffentlichkeit: Trinken in den 1970er Jahren

Diese Entwicklung intensivierte sich in den 1970er Jahren. Um 1970 weckt das Thema Frauen und Sucht darüber hinaus immer stärkeres öffentliches und politisches Interesse. Auch die BZgA steigt in die Debatte ein, wobei die Behörde zunächst vorliegende wissenschaftliche Erkenntnisse zum Thema bündelt. Sie stützt sich dabei auf die Expertise des Max-Planck-Instituts für Psychiatrie, namentlich auf deren bekannten Alkoholismusforscher Wilhelm Feuerlein.^9^ Dieser hatte sich in einem in der Fachzeitschrift *Nervenarzt* erschienenen Artikel intensiv mit dem Thema weiblicher Alkoholismus beschäftigt. Zur Lage trinkender Frauen schreibt Feuerlein, diese seien am häufigsten in Großstädten anzutreffen, stammten eher aus gehobenen sozialen Schichten, würden erst im vierten Lebensjahrzehnt behandlungsbedürftig (also deutlich später als Männer), tränken häufiger heimlich und bevorzugten stark alkoholische Getränke. Frauen tränken häufiger aufgrund von Paar- und Familienkonflikten als Männer. Letztere glichen durch ihren Alkoholkonsum nach eigenen Angaben vor allem berufliche Probleme aus und seien in ihrem Charakter eher selbstunsicher. Die Bedeutung der Ehemänner und der Ehe für die Entwicklung der Frau sei, so Feuerlein, bisher kaum untersucht worden, umgekehrt sei dies hingegen häufig geschehen.

Angeregt unter anderem durch Feuerleins Erkenntnisse führt die BzGA 1973 eine eigene Studie zum zunehmenden Alkoholkonsum von Frauen im Kontext weiblicher Berufstätigkeit durch. Die Studie arbeitet heraus, dass Ehefrauen aus der Mittel- und Oberschicht mehr Gleichberechtigung einfordern. Die daraus resultierenden Spannungen zwischen traditionellen und modernen Rollenerwartungen führten häufig zu Stress und in der Folge zu einem potenziell höheren Alkoholkonsum. Das Fazit der Studie lautet, dass Alkoholverhalten dann problematisch werde, wenn die Rollenvorstellungen der Frau den gesellschaftlichen Rollenerwartungen (von Freunden, Bezugsgruppe oder Partner) entgegenstünden. Deshalb müsse eine weitere Alkoholvorstudie vor allem nach den Erwartungen an Frauen sowie nach deren eigenen Rollenerwartungen fragen.^10^ Eine auf die Vorstudie folgende Studie und ihre Ergebnisse finden sich in den überlieferten Unterlagen nicht. Das besagt jedoch nicht, dass die Bedeutung des Themas abgenommen hätte: Parallel zu den Aktivitäten der BZgA nimmt auch in der Zeitschrift *Suchtgefahren* die Debatte über Alkoholkonsum von Frauen zu, während in den sozialpädagogischen Zeitschriften die Diskussionen eher abnehmen. Insgesamt spezialisiert sich die Alkoholismusforschung nun zunehmend und beleuchtet stärker einzelne Aspekte.

In der zweiten Hälfte der 1970er Jahre entstehen auch mehrere Monografien zum Thema Frauen und Alkohol. Drei häufig zitierte Publikationen seien hier stellvertretend vorgestellt: Herbert Riemenschneider (1976: 4), Leiter eines Fachkrankenhauses für suchtkranke Frauen, schließt an die These von Frauenalkoholismus als Folge der Emanzipation an. Er bringt dies mit der Doppel- und Dreifachbelastung durch Beruf, Haushalt und Familie in Verbindung, die er als falsch verstandene Gleichberechtigung bezeichnet. Zwei Psychologinnen betonen in ihren einschlägigen Monografien eher die Bedeutung von mit ihrer Rolle unzufriedenen, aber gleichzeitig reibungslos funktionieren-müssenden und sich an die gesellschaftlichen Erwartungen anpassenden (Haus‑)Frauen. Dabei sind sich Ilse Demel, wissenschaftliche Mitarbeiterin des Ludwig-Boltzmann-Instituts für Suchtforschung in Wien, und Marijke Mantek, Suchtreferentin bei der BZgA, allerdings uneinig über den sozioökonomischen Status der betroffenen Frauen. Während Demel (1976: 9, 24f.) den niedrigen Bildungsstand eines großen Teils der Alkoholikerinnen betont, die aus zerrütteten Familien stammten, arbeitet Mantek (1979) heraus, dass eher Frauen aus der Oberschicht Alkohol konsumierten. Der Alkoholkonsum nehme ohnehin mit dem sozialen Status zu, daher sei dieser ihrer Beobachtung gemäß entscheidender als das Geschlecht. Mantek zufolge legen die vorhandenen Studien den Schluss nahe, dass trinkende Frauen eher maskulin orientiert seien und der Alkoholkonsum ihnen beim Ausleben dieser Orientierung helfe (ebd.: 30–36). Deutliche Unterschiede gibt es laut Demel (1976: 8) allerdings bei der Einstellung der Bevölkerung zu männlichem und weiblichem Trinkverhalten: Ersteres würde toleriert, Letzteres hingegen geächtet.

Die zunehmende Aufmerksamkeit für trinkende Frauen auch in populären Medien sowie die Rezeption der einschlägigen Forschung in den 1970er Jahren verdeutlichen auch, wie widersprüchlich die unterschiedlichen wissenschaftlichen Erklärungsweisen zum Teil sind. Dabei zielen die Zeitschriftenartikel weniger darauf, empirische Sachverhalte aufzudecken, als die Widersprüche und sich daraus ergebende gesellschaftliche Folgen des Alkoholkonsums zuzuspitzen. Es entsteht der Eindruck, dass die Autor:innen auf diese Weise die Aufmerksamkeit auf dieses spezielle Thema lenken wollten. Konsens scheint in den populären Schriften zu sein, dass die „Luxusverwahrlosung“ für die ständige Zunahme alkoholkranker Frauen verantwortlich sei.^11^ Widersprüchlicher ist hingegen die Rolle, die der Emanzipation für den zunehmendem Alkoholkonsum zugeschrieben wird: „Macht Emanzipation die Frauen krank?“, fragt etwa *Der Spiegel.*^12^. Für die *Augsburger Allgemeine*^13^ animiert hingegen gerade das Scheitern der Emanzipationsbestrebungen Frauen zum Trinken. Uneinigkeit herrscht auch darüber, wodurch die Frauen krank geworden sind: durch die zunehmende Erwerbstätigkeit, wie die *Morgenpost*^14^ anmerkt, durch die Doppelbelastung von Familie und Beruf, wie der *Mannheimer Morgen*^15^ konstatiert, oder durch die eintönige Routine des Hausfrauenalltags, wie der *Stern*^16^ hervorhebt? Für die *Neue Hannoversche Presse* ist diese Unterscheidung eigentlich egal: Sowohl das Trinken von Hausfrauen als auch der „Feierabendwein“ berufstätiger Frauen seien jeweils eine Seite derselben Medaille – des „Wohlstands-Suffs“.^17^

Bis Ende der 1970er Jahre liegen zahlreiche wissenschaftliche Studien zur Alkoholabhängigkeit von Frauen vor, die daraus resultierenden Erkenntnisse sind jedoch widersprüchlich, da sie genutzt werden können, um die jeweils eigene Weltanschauung zu untermauern. Die Tagespresse vermittelt die verschiedenen Forschungsergebnisse der breiten Bevölkerung. Um die sich wandelnde Bedeutung des weiblichen Alkoholkonsums zu verstehen, hilft ein Blick auf die sich im Untersuchungszeitraum verändernden Lebensbedingungen von Frauen. Gerade die rechtliche Stellung der Frauen verändert sich im Lauf der 1970er Jahre stark. So ist es Frauen leichter möglich, sich von ihren Ehepartnern scheiden zu lassen, da 1977 mit der Reform des Ehe- und Scheidungsrechts das Zerrüttungsprinzip die Schuldfrage ersetzt. Gerade für geschiedene Ehefrauen hat dieses Urteil weitreichende Konsequenzen, da noch in den 1960er Jahren in knapp 40 Prozent der Fälle die Frauen „schuldig geschieden“ wurden und dadurch ihren Unterhaltsanspruch verloren (Neumaier 2019: 382). Zudem wird die Gewalt gegen Frauen in den 1970er Jahren von der Frauen- und Frauenhausbewegung thematisiert. Seit Ende der 1970er Jahren bieten Frauenhäuser Frauen Schutz, die vor den – nicht selten im Alkoholrausch begangenen – Gewaltexzessen ihrer Partner fliehen (ebd.: 414–416).

Nicht nur das Thema der männlichen Gewalt als Folge von Alkohol, sondern auch der Frauenalkoholismus wird von der Frauenbewegung diskutiert. Allerdings wird das Phänomen anders eingeordnet – nämlich als Reaktion von Frauen auf patriarchale Dominanzansprüche, wie unter anderem ein Schwerpunktheft der Zeitschrift *Courage *zeigt. Darin sind mehrere Berichte betroffener Frauen abgedruckt, die von ihrem Weg in die Sucht sowie von ihrem Leben mit dieser berichten (Anonymus 1979a: 19–24). Auch Partnerinnen trinkender Frauen kommen zu Wort (Anonymus 1979b: 30–33). Schließlich bietet die Zeitschrift den Frauen einen „Selbsttest“ an, der auf einem Modell verschiedener Phasen des Trinkens vom Alkoholismusforscher Elvin Morton Jellinek beruht. Dieses unterscheidet zwischen einer Vorstufe, einer kritischen Phase und der letzten Phase – dem chronischen Alkoholismus. Die Leser:innen werden aufgefordert, sich im Selbsttest in das Schema einzuordnen (Anonymus 1979c: 25). Damit schließen die Feminist:innen an den hegemonialen wissenschaftlichen Diskurs an, der sich zentral an Jellinek orientiert. Unter anderem berät Jellinek seit den 1950er Jahren die Weltgesundheitsorganisation (WHO) in Fragen des Alkoholismus. Er vertritt schon früh den Ansatz, dass es sich bei Alkoholismus um eine Krankheit handelt und grenzt sich damit von anderen Forscher:innen ab, die Alkoholsucht eher als Willensschwäche begreifen. In einer Monografie führt Jellinek die später berühmt gewordene Typologie von Alpha‑, Beta‑, Gamma- und Delta-Trinkern ein, die er ausschließlich anhand männlicher Probanden entwickelt (Jellinek 1960). Die *Courage* nimmt Jellineks Alkoholismus-Selbsttest offenbar kritiklos auf, anstatt ihn als patriarchal und pathologisierend zu kritisieren. Auch die Zeitschrift *Emma* widmet sich Ende der 1970er Jahre dem Thema Alkoholismus: Die Autorinnen eines diesbezüglichen Artikels betonen, dass das Phänomen oft gut abgesicherte Frauen zwischen 40 und 50 Jahren betreffe, die durch traditionelle Geschlechtervorstellungen dazu verdammt würden, ein Hausfrauendasein zu fristen, das sie nicht auslaste. Als „grüne Witwen“ säßen sie verlassen in der naturnahen Vorstadt in Bungalows und betäubten ihre Einsamkeit mit Alkohol – und oft auch mit Tranquilizern (Pinkus & Müller 1977a: 36–39). Um diese These zu untermauern, druckt die *Emma* ein Interview mit einer als Trinkerin bezeichneten Frau ab. Diese schildert ihre Freude an der Berufsarbeit, die aber mit ihrer Heirat und der Geburt ihres Kindes abrupt geendet habe. Auch als das Kind in den Kindergarten kam, habe ihr Ehemann ihr eine Rückkehr in den Beruf verwehrt. Daraufhin habe sie sich die einsamen Nachmittage mit mehreren Cognacs zum Kaffee schöngetrunken, was schließlich in eine Sucht mündete (Pinkus & Müller 1977b: 40f.).

**Abb. 3:**
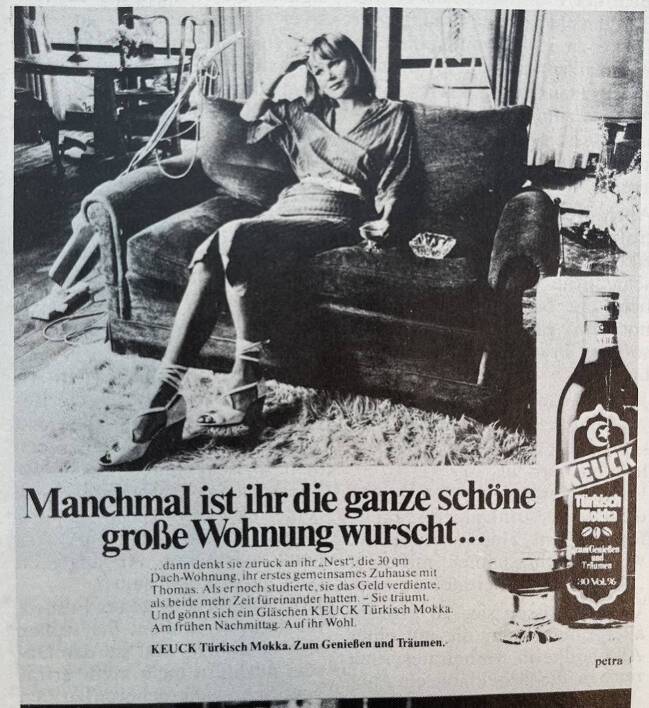
Manchmal ist ihr die ganze schöne große Wohnung wurscht (Pinkus & Müller 1977a: 38)

Illustriert mit der Abb. 3 veröffentlicht die Zeitschrift *Emma* einen Fragebogen, anhand dessen die Leser:innen testen können, ob sie eine Alkoholabhängigkeit entwickelt haben (Pinkus & Müller 1977a: 39). Offenbar verstehen es Feminist:innen, den Begriff des Alkoholismus, der zu diesem Zeitpunkt von sozialen Bewegungen bereits mit psychosozialem Wissen gefüllt werden kann, für sich zu nutzen. Sie versprechen sich davon, so ließe sich vermuten, einen Gewinn für emanzipatorische Anliegen von Frauen. Gleichzeitig stehen die feministischen Kritiken in *Courage* und *Emma* jedoch beispielhaft für einen sich wandelnden Umgang mit weiblichem Alkoholkonsum. Um 1980 erreicht die Debatte um die Alkoholabhängigkeit von Frauen einen neuen Höhepunkt. Während männliche Forscher den zunehmenden Alkoholkonsum von Frauen weiterhin als Folge von Emanzipationsbestrebungen beschreiben, skandalisieren feministische Professionelle diese Erklärungsweise nun lautstark.

## „Die beste Alkoholprävention ist die Antiemanzipation“: Die Konferenz der DHS und ihre Folgen

1980 ist die Debatte um den weiblichen Alkoholkonsum so prominent, dass die DHS sich dazu veranlasst sieht, eine zweite Konferenz zum Thema zu veranstalten. Diesmal weitet sie die Beschäftigung aber auf das Thema „Frauen und Sucht“ aus. Das Vorwort des Tagungsbandes konstatiert, dass sich die Lage inzwischen zugespitzt habe (Anonymus 1981). Um die Bedeutung der sich wandelnden Frauenrollen für die Entwicklung von Abhängigkeiten zu untersuchen, lädt die DHS mehrere in ihrem Fachgebiet bekannte Forscher – allesamt Männer – ein, um das Thema aus psychiatrischer, sozialwissenschaftlicher und phylogenetischer, ergo stammesgeschichtlicher Sicht zu kommentieren. Klaus Wanke, Direktor der Universitätsnervenklinik in Homburg, schildert aus psychiatrischer Sicht, trinkende Frauen sprächen schlechter auf Therapien an, insbesondere auf Gruppentherapien (Wanke 1981). Der Soziologieprofessor und bekannte Familienforscher Gerhard Wurzbacher wiederholt die These von angeblich besonders empfindsamen Frauen, die durch die Auflösung traditioneller Familienstrukturen stark verunsichert seien (Wurzbacher 1981). Dabei schloss er an sein Werk *Leitbilder des deutschen Familienlebens* an, das 1950 erstmals erschien und bis Ende der 1960er Jahre wiederholt aufgelegt wurde. Das von ihm beobachtete, zunehmend partnerschaftliche Ehemodell sah er bereits dann gegeben, wenn die Ehefrau ihre Hausfrauentätigkeiten weitgehend selbstständig ausführen konnte (Neumaier 2019: 320; Wurzbacher 1969).^18^ Wolfram Keup, Psychiater und ärztlicher Direktor der Karl-Bonhoeffer-Nervenklinik in Berlin, erklärt die Unterschiede im Suchtverhalten von Männern und Frauen hingegen im Wesentlichen stammesgeschichtlich, indem er sie aus der unterschiedlichen körperlichen Ausstattung ableitet. Die geschlechtsspezifischen Unterschiede hält Keup daher nicht für ohne Weiteres veränderbar. Er konstatiert, dass diese Unterschiede bis tief in die Psyche hineinreichen und spitzt weiter zu:

„Verlangt man vom Menschen eine Emanzipation „auch in diesem Sinne“, so verlangt man vom ihm das Verlassen einer ontogenetisch nachvollzogenen phylogenetischen Lebensbasis im Inneren und schafft damit unauflösbare Konflikte“ (Keup 1981: 103).

Keups zugespitzte Formulierung bringt die Spannungen zwischen den verschiedenen Deutungen auf den Punkt: Auf der einen Seite gelten emanzipatorische Bestrebungen als suchterzeugend, auf der anderen als abhängigkeitsreduzierend. Die Konzentration der Debatte auf vermeintliche Defizite von Frauen sorgt auf der Konferenz im Anschluss an die Plenumsvorträge für kontroverse Diskussionen. Die mehrheitlich von Frauen besuchte Arbeitsgruppe „Biographie und Suchtentwicklung“ kritisiert, dass sich das Frauenbild der männlichen Redner durch Passivität, Demut und Bescheidenheit sowie durch Erwartungen an emotionale Zuwendung und Opferbereitschaft auszeichne. Unbeantwortet bliebe dabei die Frage, ob die Doppelbelastung von Frauen diese nicht eher vor dem Trinken bewahre. Durch die als selektiv wahrgenommene Auswahl der Referenten seien feministische und gewerkschaftliche Positionen gar nicht erst diskutiert worden (Vogt 1981). Zudem könne, so eine weitere Kritik, weibliche Emanzipation nicht auf Berufstätigkeit, Doppelbelastung und eine Imitation männlicher Verhaltensweisen reduziert werden. Vielmehr sei die Befreiung aus Abhängigkeit eine lebenslange Aufgabe. In diesem Sinne schlössen sich Emanzipation und Abhängigkeit gegenseitig aus (Schönherr 1981: 164).

Die auf der Konferenz vorherrschende androzentrische Sichtweise kritisierte auch eine weitere Arbeitsgruppe mit dem Titel „Thema verfehlt“, die spontan von Teilnehmerinnen gebildet worden war. Bei dem Kongress handle es sich, so die beteiligten Frauen, um eine Veranstaltung von Männern für Männer, die sich gegenseitig in dem bestätigen würden, was sie ohnehin schon über Frauen und süchtiges Verhalten dächten:

„Man hat uns in ungezählte Statistiken gepresst, uns für psychisch instabil und depressiv erklärt, mit niedriger Frustrationstoleranz ausgestattet, für nicht gruppentherapiefähig und für biologisch und genetisch prädestiniert für den Suchtmittelmissbrauch beschrieben – all das immer am ungebrochenen Maßstab Mann gemessen und zu ihm in Relation gesetzt. […] Wir sollen Nestwärme schaffen, um die Mutterhocker zur Entfaltung zu bringen, aber wir sollen nicht auf die Idee kommen, die Kälte und emotionale Verarmung um uns herum mit Alkohol, Heroin und Tabletten zu kompensieren“ (Kreyssig & Kurth 1981: 165).

Mit deutlichen Worten fordern die Teilnehmerinnen der Arbeitsgruppe von den (männlichen) Wissenschaftlern, sich damit zu beschäftigen, von welchen Zwängen und Belastungen Frauen befreit werden müssten, um ihnen ein unabhängiges Leben in der Gesellschaft zu ermöglichen. Dass die bisherige mediale Berichterstattung dermaßen negativ sei und Frauenemanzipation für die Sucht verantwortlich mache, sei die Folge einer misogynen Forschungstradition. Die Forscherinnen spielen hier offensichtlich auf den erwähnten *Spiegel*-Artikel an, der fragt, ob die Emanzipation krank mache.^19^

Die Presseberichterstattung zur Konferenz stürzt sich zunächst auf die Kontroverse: „‚Listige‘ Frauen buhten Professor aus. Suchtgefahren mit mangelnder Beschäftigung im modernen Haushalt erklärt“, ätzt etwa die *Westdeutsche Allgemeine* und berichtet, dass Rednerinnen dem kritisierten Wolfram Keup rieten, sich mit „Emanzipationslektüre“ zu befassen, um sein reaktionäres Denken zu überwinden.^20^ „Die Wissenschaftler wissen nicht, was das Weib will“, unkt das *Spandauer Volksblatt Berlin-West *und spielt damit auf die feministische Kritik an den männlichen Interpretationen weiblicher Sucht an. Der Artikel berichtet, dass die Frauen auf der Konferenz mehr Möglichkeiten für Teilzeitarbeit und eine stärkere Beteiligung von Vätern an der Sorgearbeit gefordert hätten. Dies entlaste Frauen am besten und diene so auch der Alkoholismusprävention.^21^ Die *Tageszeitung* hält in ihrem Bericht über die misogynen Vorträge fest, dass sich die massive Kritik von Feministinnen am Kongress schließlich nicht mehr habe befrieden lassen. Das habe zur Gründung einer ständigen, an die DHS angegliederten Arbeitsgruppe „Frauen und Sucht“ geführt, die sich von nun an kritisch mit dem Thema beschäftigen wolle.^22^

Zwei radikale Kritikerinnen, die Diplompädagogin Ulrike Kreyssig und die Psychologin Anne Kurth, meldeten sich kurz nach der Konferenz in der sozialpädagogischen Zeitschrift *Sozialmagazin* noch einmal zu Wort. Dort versuchen sie, das süchtige Verhalten von Frauen anders zu erklären.^23^ Sie stellen dabei nicht infrage, dass die Zahl abhängiger Frauen zugenommen hat. Eine an den Interessen von Frauen ansetzende Beratungsarbeit müsse sich aber die Aufgabe stellen, die in der Sucht zum Ausdruck kommende Form selbstzerstörerischer Verweigerung gesellschaftlicher Unzumutbarkeiten konstruktiv nach außen zu wenden. Das hieße zunächst, Frauen in ihrem Selbstfindungsprozess zu unterstützen und sie darin zu bestärken, Nein zu sagen und Verantwortung an Männer zu delegieren. Solange Letztere ihren Männlichkeitswahn aber nicht ablegten, bliebe den Frauen kaum etwas anderes übrig, als sich süchtig zu verhalten.^24^ Sybille Plogstedt fasst die gesellschaftliche Debatte in einem Artikel für den Deutschen Frauenrat zusammen. Frauen würden sich weiterhin dem patriarchalen System unterwerfen, was sich auch in den Suchtstatistiken abbilde: Nicht die Emanzipation treibe Frauen in die Sucht, sondern die Anpassung an soziale Normen.^25^ Die geschilderten feministischen Interventionen kennzeichnen den Beginn einer Anfang der 1980er Jahre einsetzenden Wende: Frauen versuchen nun selbst, die Gründe für weibliches Trinken zu beschreiben und unterschiedliche Wege in die Sucht nachzuzeichnen. Dabei grenzen sie sich keineswegs von pathologisierenden Begriffen wie „Alkoholabhängigkeit“ ab, sondern nutzen diese, um patriarchale Gewaltverhältnisse zu thematisieren und separate Räume für Frauen zu fordern.

## Trinken und Emanzipation: Alkoholismus als Scheitern an gesellschaftlichen Gewaltverhältnissen

1984 veranstaltet die feministische Arbeitsgruppe, die sich auf der erwähnten DHS-Konferenz zu „Frauen und Sucht“ zusammengefunden hat, eine eigene Tagung. Es entsteht eine Dokumentation, die sich laut den Herausgeberinnen der Frage widmet, wie eine frauengerechte Beratung und ein gutes Leben für Frauen aussehen könnten. Dabei bezeichnen sie auch die Sehnsucht nach Liebe und ausschließlicher Bindung an eine Person als abhängigkeitserzeugend (Merfert-Diete & Soltau 1984: 9f.). Um Frauen von Süchten zu befreien, müsse man sie unterstützen, emotionale Abhängigkeiten und erlernte Passivität zu überwinden (Soltau 1984). Die erfahrene Abhängigkeit setze sich als sogenannte Co-Abhängigkeit fort, mit der Frauen ihre eigene Unfreiheit verfestigten, indem sie ihre trinkenden Männer umsorgten und deren Konsum erst ermöglichten, anstatt ihre eigenen Lebensbedingungen zu verbessern (Walcker-Meyer 1984: 80–82).

Mitte der 1980er Jahre entsteht auch Selbsthilfeliteratur zum Thema trinkende Frauen. In der Biografie *Ich heiße Erika und bin Alkoholikerin* schildert die Autorin Heide Nullmeyer (1980) den Weg einer Frau vom Alkoholkonsum bis zum Entzug: Sie beschreibt, wie „Erika“ zunächst ihren trinkenden Ehemann unterstützt, dabei selbst immer mehr Alkohol konsumiert, um ihr Leid zu ertragen, ihre eigene Sucht aber verleugnet und in der Klinik ankommend weiterhin behauptet, sie sei nur eine mittrinkende Angehörige, aber keinesfalls selbst abhängig geworden. Der von Antje Doll (1992) herausgegebene Sammelband *Endlich reden. Frauen von alkoholabhängigen Männern berichten* lässt in Interviews die Angehörigen zu Wort kommen. Auf deren Basis stellt Doll die These auf, Frauen in Partnerschaften mit alkoholkranken Männern würden oft über ihre zentralen Bedürfnisse schweigen und die Diskrepanzen aushalten, um die Beziehung nicht zu gefährden. Gleichzeitig hinterfragten sie oftmals die Sucht ihrer Partner nicht und würden so zu Co-Abhängigen.

Auch wissenschaftliche Publikationen widmen sich in den 1980er Jahren dem Zusammenhang zwischen Partnerschaft, Co-Abhängigkeit und dem Alkoholkonsum von Frauen. Die Forschung beschäftigt sich zudem mit der Bedeutung der Familie für die Entstehung und Aufrechterhaltung von Sucht. Diese Richtung der Familienforschung knüpft an die Debatte um die Co-Abhängigkeit an und geht von der Idee der Familie als sozialem System aus. In diesem seien Abhängige das Symbol eines familiären Nicht-Funktionierens. Dabei halte die Familie zusammen – koste es, was es wolle –, um einen weiteren Kollaps zu vermeiden. Für den sozialen Kitt sorgten meistens die (Ehe‑)Frauen (Hallmeier 1985). Familientherapien konzentrieren sich dagegen meist auf die Stärkung der positiven Kommunikation (Reichelt-Nauseef & Hedder 1985). Schließlich fokussiert sich die Forschung auf die Wege von Frauen in die Sucht. Eine kritische Auseinandersetzung mit Suchtmodellen stammt von der Psychologin Irmgard Vogt, die später Professorin für Beratung in der Sozialen Arbeit mit Schwerpunkt Suchthilfe sowie Direktorin des Instituts für Suchtforschung an der Fachhochschule Frankfurt am Main wird. Sie konstatiert, dass bisher bekannte Suchtmodelle – beispielsweise jene von Jellinek und Feuerlein – an alkoholkranken Männern entwickelt worden sind. Dabei fielen frauenspezifische Besonderheiten wie das Schwanken zwischen hohem Konsum und Abstinenz oder die Verbindung zwischen Alkoholkonsum und Essstörungen unter den Tisch. Vogt unterscheidet aufgrund ihrer eigenen Erhebung vor allem zwei Arten von Trinkerinnen: Die erste Gruppe seien Frauen, die schon in ihrer Kindheit mit Alkoholkonsum konfrontiert worden seien und daher selbst schon früh zu trinken begonnen hätten. Diese Frauen bemühten sich sehr um Liebe und Zuneigung von anderen. Als Kinder seien sie oft körperlich und sexuell missbraucht worden. Die zweite Gruppe von Alkoholikerinnen fange erst im Alter von 30 Jahren an zu trinken und wechsle relativ schnell von einem moderaten zu einem auffälligen Konsum – oftmals wegen Einsamkeit, kritischen Lebensereignissen oder Verlusterfahrungen. Infolge dieses Konsums komme es dann häufig zu Gewalt in der Beziehung. Diese Frauen lebten zwar in geordneten Verhältnissen, seien aber in Lebenskrisen auf sich allein gestellt. Nicht selten hätten sie auch psychosomatische Beschwerden, die durch den Alkoholkonsum zurückgedrängt würden. Außerdem seien sie meist erfolgreich im Beruf (Vogt 1987).

Die besondere Verbindung zwischen Alkoholismus und erfahrener Gewalt betonen auch frauenspezifische Einrichtungen, die zu Beginn der 1980er Jahre entstehen. Insbesondere die 1981 gegründete Berliner Einrichtung *Die Zwiebel*, die sich ausschließlich an medikamenten- und alkoholabhängige Frauen wendet, sowie das 1983 gegründete Berliner Projekt *Violetta Clean*, das sich allgemein drogenabhängigen Frauen öffnet, weisen auf mangelnde Therapieangebote für Frauen im Suchtbereich hin. Sie versuchen damit das Problem zu lösen, dass sich suchtgefährdete Frauen seltener in gemischtgeschlechtlichen Einrichtungen aufhalten, weshalb diese sich mit ihren Angeboten eher an Männern orientierten. Zudem müssten Frauen in klassischen Trinkerasylen wiederholt Gewalterfahrungen machen:

„Ganz pauschal gesagt, waren in den Trinkerasylen Männer unter sich mit der Folge, daß die Umgangsformen dort ebenso rau und ruppig waren wie in anderen Männerbünden und -vereinen auch, z. B. dem Militär […]. Ganz entsprechend gab der Macho-Mann für lange Zeit den Ton an in der Suchtkrankenhilfe, und das galt ebenso für die Alkoholiker wie für die Helfer“ (Vogt 1989: 60).

Als Gegenbewegung zu den klassischen Asylen versuchen die neuen Projekte, konsumierenden Frauen einen Raum für sich selbst, für die Entfaltung ihrer Bedürfnisse und zur Entwicklung ihrer Autonomie anzubieten. Die Frauen sollen befähigt werden, handwerkliche und finanzielle Dinge selbst zu erledigen, damit sie sich nicht in heterosexuelle Abhängigkeitsbeziehungen begeben, nur um versorgt zu sein (Benesch-Daugs & Falke-Roos 1984). Die Initiatorinnen der Projekte reagieren damit auch auf eine Situation, in der zwar die Zahl süchtiger Frauen kontinuierlich steigt, es aber (außer der bereits referierten) kaum Literatur gibt, die diese Zunahme aus der Sicht von Frauen erklärt, geschweige denn geschlechtsspezifische Angebote macht. Bund und Länder, so beklagen etwa Kurth & Kreyssig (1984), sähen weder den Bedarf für frauenspezifische Suchteinrichtungen, noch initiierten sie deren verlässliche Finanzierung oder eine begleitende Forschung (vgl. auch Hentschel 1986). Dabei gäbe es, wie feministische Autorinnen nicht müde werden zu betonen, kein wirksameres Mittel gegen die weibliche Abhängigkeit als die Emanzipation (Schönherr 1984). Frauenspezifische Suchtarbeit müsse daher darauf zielen, „daß die Bekämpfung von Abhängigkeit sich nicht allein auf die Abhängigkeit von Suchtmitteln beschränken darf, sondern alle ihre Formen auf allen Ebenen unseres Lebens erfassen muss“ (ebd.: 224).

## Zwischen Abhängigkeit, Unabhängigkeit und Co-Abhängigkeit – ein Fazit

1989 widmet sich die DHS mit einer großen Konferenz erneut dem Thema Sucht und Geschlecht. Diesmal nehmen laut Veranstalterin erstmals mehr Frauen als Männer teil (DHS 1990: 11–13). Offensichtlich sind die Verantwortlichen bemüht, einen ähnlichen Eklat wie auf der Konferenz von 1980 zu vermeiden. Wie Senta Feselmayer und Wolfgang Beiglböck in einem der Hauptvorträge ausführen, habe nicht die Emanzipation in den letzten Jahrzehnten zu einer erhöhten Zahl an Suchtabhängigen geführt. Vielmehr seien die Bedingungen, unter denen weibliche Unabhängigkeitsbestrebungen durchgesetzt werden, dafür verantwortlich: Diese hätten zu einer größeren Rollenunsicherheit und zu starken Sanktionen bei Nichterfüllung tradierter Geschlechternormen geführt (Feselmayer & Beiglböck 1990: 30). Dieses späte Umdenken ist der vorläufige Endpunkt einer Debatte zum Frauenalkoholismus, in der um Besonderheiten weiblichen Trinkverhaltens gerungen wird. Doch wie haben sich durch diese Debatte die Differenzkonstruktionen von männlich/weiblich sowie normal/verrückt verschoben? Welche Rolle spielen in diesem Prozess Psychologie und Pädagogik, gerade im Verhältnis zur Psychiatrie?

Die historische Rekonstruktion der Debatte über trinkende Frauen liefert einen wichtigen Baustein zur noch kaum erforschten Geschichte des Frauenalkoholismus in der zweiten Hälfte des 20. Jahrhunderts. Die Debatte ist dadurch gekennzeichnet, dass sich die gesellschaftliche Rolle von Frauen verändert und patriarchale Gegenbewegungen darauf reagieren. Seit den 1950er Jahren und insbesondere im Zuge der Neuen Sozialen Bewegungen seit 1968 gerät der Alkoholkonsum von Frauen erstmals ins Visier der Forschung. Dieser wird hauptsächlich als Folge einer zusammenbrechenden Gesellschafts- und Geschlechterordnung beschrieben – wahlweise in der Figur des einsamen „trinkenden Karriereweibs“ oder des Opfers technisierter und entseelter Hausfrauenarbeit. Noch bis in die 1970er Jahre werden Ehefrauen für die Trunksucht ihrer Männer verantwortlich gemacht. Von den Frauen wird verlangt, für die Abstinenz ihrer Ehegatten zu sorgen und ihnen ein gemütliches Heim zu schaffen. Gleichzeitig sind spätestens seit den 1980er Jahren Frauen zu Co-Abhängigen erklärt worden, wenn sie ihrem Partner das Leben allzu behaglich machen. Gegen diese Behauptungen setzen sich insbesondere in den 1980er Jahren Feminist:innen zur Wehr: Sie entlarven die männlich dominierten Diskussionen über Frauenalkoholismus als Versuch, die traditionelle Geschlechterordnung wiederherzustellen. Dagegen beharren sie darauf, dass die Alkoholabhängigkeit von Frauen nur durch einen Abbau klassisch patriarchaler Abhängigkeitsbeziehungen zu lösen sei, in denen Frauen zum großen Teil immer noch leben würden.

Mit der (Nachkriegs‑)Geschlechterordnung, die sich im Diskurs über Frauenalkoholismus artikuliert, verändert sich zugleich die Grenzziehung zwischen normalem und pathologischem Alkoholkonsum. Die in den 1950er Jahren eingeführte medikamentöse Antabus-Kur bringt nicht die erwünschten Erfolge. Wenngleich weiterhin medikamentöse Therapien insbesondere beim Alkoholentzug eingesetzt werden, sind medizinische Behandlungen weniger dominant. Infolgedessen gewinnen moralisch-erzieherische Belehrungen durch Fürsorge und Theologie, die schon seit dem 19. Jahrhundert in der Alkoholikerfürsorge eingesetzt wurden, erneut an Gewicht. Deren Grundlage war eine Beschreibung von Trunksucht als einer Krankheit des Willens. Diese Entpathologisierung der Trunksucht setzt Ende der 1950er Jahre ein und wird von restriktiven erzieherischen Maßnahmen begleitet. Bereits Ende der 1960er Jahre folgt allerdings eine (Re‑)Pathologisierung der Sucht – mit paradoxen Effekten: Wie Valverde (1998) betont, wird Alkoholabhängigkeit Ende der 1960er Jahre zwar als Krankheit eingestuft, es gibt dafür aber kaum eine medizinische Behandlung. Deshalb verzichten Psychiater:innen auf eine weitere Pathologisierung der Alkoholabhängigkeit (ebd.: 11, 99). Der mangelnde Erfolg medizinischer Behandlungsansätze bei gleichzeitiger Überfüllung der Anstalten durch Alkoholabhängige bedingen, dass psychologische und sozialpädagogische Ansätze sowie neue Ansätze der Selbsthilfe entwickelt und artikuliert werden können (ebd.: 106).^26^ Psycholog:innen und Sozialpädagog:innen liefern mit einem vorwiegend biografischen Ansatz neues Erklärungswissen, ohne dabei jedoch das Krankheitskonzept grundsätzlich zu hinterfragen. Dies zeigt sich auch darin, dass die Fürsorge/Soziale Arbeit in der Nachkriegszeit auf psychologisches, psychiatrisches und psychoanalytisches Wissen zurückgreift, das auch in der Ausbildung beziehungsweise dem Studium eine große Rolle spielt. Auch Publikationsorgane der Frauenbewegung wie die Zeitschriften *Emma* und *Courage* grenzen sich keineswegs vollständig vom neuen Begriff der Alkoholabhängigkeit ab. Vielmehr bieten sie ihren Leser:innen Möglichkeiten, ihre eigene Alkoholabhängigkeit mithilfe eines Fragebogens selbst einzuschätzen und gegebenenfalls neue psychosoziale Angebote für sich zu nutzen. Um 1970, also etwa zeitgleich mit der (Re‑)Pathologisierung, setzt in der Debatte um Alkoholkonsum eine sozialpädagogisch-psychologische Wende ein, die psychiatrisches Wissen zwar nicht gänzlich ersetzt, den Suchtbegriff jedoch stark erweitert. Diese Neuausrichtung ermöglicht eine Aneignung des Begriffs Alkoholabhängigkeit auch durch die Frauenbewegungen. Dies zeigt sich sowohl bei den Debatten auf der DHS-Konferenz von 1980 als auch bei den in der Folge initiierten Frauenprojekten. Die beschriebene Entwicklung lässt sich auch als eine Erosion von Normalität und Verrücktheit beschreiben. Bezogen auf Alkoholismus bedeutet das gerade nicht, dass die Abhängigkeit entpathologisiert wird. Aufgrund des eingeschränkten Erfolges medizinischer Behandlungsversuche erodiert jedoch der klassische Suchtbegriff. Diese Lücke kann psychosoziales Erklärungswissen füllen. Neue Professionen wie Psychologie und Sozialpädagogik, aber auch neue Bewegungen wie die Frauenselbsthilfe können sich dieses Wissen aneignen und es neu interpretieren.

Der neue Begriff vom pathologischen Alkoholkonsum eröffnet Frauen letztendlich Handlungsspielräume: Die inzwischen allgemein anerkannte Erklärung, dass Frauen aufgrund gesellschaftlicher Entwicklungen substanzabhängig und damit psychisch krank werden, erlaubt es ihnen, diese Umstände zu skandalisieren und psychosoziale Hilfen einzufordern. Die reale Zunahme des Alkoholkonsums ist jedoch auch Teil jenes Freiraums, den sich Frauen durch emanzipatorische Kämpfe erobert haben. Die trinkende Frau ist somit Teil des Paradoxons von Freiheit in der Unfreiheit geworden. Sie kann die gesellschaftlichen Ungleichheiten zwar nicht überwinden, diese erlauben es ihr aber wenigstens, sich mit Alkohol zu betäuben.

## Footnotes

1 *Erster Jahreskongress Frauen und Sucht: Thema verfehlt. Die Tageszeitung*, 03.11.1980, ohne Seitenangabe, in: Berliner Frauenforschungs-, -bildungs- und -informationszentrum (FFBIZ), Rep14a, 1976–2003. Die im Folgenden aus dem FFBIZ angegebenen Archivalien geben z. T. Seitenzahlen und andere wichtige Informationen nicht an. Die Angaben wurden so aus dem Bestand übernommen.

2 Erste Analysen haben ergeben, dass es die Diskurse um einen zunehmenden Alkoholkonsum der Frauen – unabhängig von einer Zunahme realer Zahlen – in der DDR in dieser Form nicht gegeben hat (vgl. Balz 2020; Linnek 2016: 212f.; Barsch 2009: 160f.).

3 Letztere wurden über Pubmed recherchiert.

4 Bei den Archivalien des FFBIZ handelt es sich im Wesentlichen um eine systematische Zeitungs- und Zeitschriftensammlung zum Thema Frauen und Alkohol. Ich danke Susanne Doetz für den Hinweis auf diesen Quellenbestand.

5 Zum Begriff des Gegenwissens vgl. den Beitrag von Susanne Doetz in diesem Heft.

6 Bundesgerichtshof, Urteil IV ZR 246/63 vom 07.10.1964.

7 Bundessozialgericht, Urteil 3RK 63/66 vom 18.06.1968.

8 Grunenberg, Angelika 1967. Arme Kreaturen, die niemand will. Alkohol – eine Gefahr für Alleinstehende Frauen. [unbekannte Zeitung], 02.09.1967, in FFBIZ: Arep400; BRD14a, verschiedene Suchtprobleme, 1976–2003.

9 Zur wiederholten Zusammenarbeit der BzGA mit dem Max-Planck-Institut für Psychiatrie vgl. Balz (2024).

10 Entwurf eines Untersuchungsansatzes zum Thema Alkoholkonsum von Frauen vom 31.07.1973, in BArch 310/1972.

11 Trinkerinnen schätzen härtere Sachen. *Frankfurter Rundschau*, 20.10.1973, in FFBIZ, Arep400 BRD14a, 1976–2003.

12 Macht Emanzipation die Frauen krank? *Der Spiegel, *05.05.1975, in FFBIZ, ARep400, 1970–1984.

13 Der Alkohol ersetzt den Partner. *Augsburger Allgemeine, *04.11.1977, in FFBIZ, ARep400, 1970–1984.

14 Frauen und Wein. *Morgenpost,* 19.11.1978, in FFBIZ, Arep400 BRD14a, 1976–2003.

15 Kirsch-Meyer, Waltraud 1978. Der Griff zur Flasche nimmt bei Frauen zu. *Mannheimer Morgen*, 16.01.1978, in FFBIZ, ARep400, 1970–1984.

16 Kroll, Benno 1975. Betrunken am Kochtopf. *Stern,* 04.12.1975, in FFBIZ, Arep400 BRD14a, 1976–2003.

17 Wohlstands-Suff nimmt zu. Wenn Frauen zur Flasche greifen. *Hannoversche Presse*, 17.03.1979, in FFBIZ, ARep400, 1970–1984.

18 Wurzbacher war Mitglied der NSDAP und arbeitete in den 1940er Jahren als Geschichtslehrer an Adolf-Hitler-Schulen und auf verschiedenen SS-Ordensburgen. Von 1958 bis 1973 war er beratend für das Familienministerium tätig. In der 2010 publizierten Gesamtdarstellung seines Werks bleibt das Thema „Frauen und Sucht“ ausgespart (Schmidt & Krieger 2010).

19 Siehe Anmerkung 12.

20 Schöneberg, Rita 1980. „Listige“ Frauen buhten Professor aus. Suchtgefahren mit mangelnder Beschäftigung im modernen Haushalt erklärt. *Westdeutsche Allgemeine Zeitung*, 30.10.1980, in FFBIZ, ARep400, 1970–1984.

21 Die Wissenschaftler wissen nicht, was das Weib will. *Spandauer Volksblatt Berlin-West*, 31.10.1980, in FFBIZ, ARep400, 1970–1984.

22 Jahreskongress Frauen und Sucht: Thema verfehlt. *Die Tageszeitung*, 03.11.1980, in FFBIZ, Arep400 BRD14a, 1976–2003.

23 Zu den wissenschaftlichen Veröffentlichungen zum Thema weiblicher Sucht vgl. u. a. Kreyssig & Kurth (1984). Ulrike Kurth war zunächst als wissenschaftliche Mitarbeiterin an der FU Berlin (1980–1982) tätig und seit 1982 an der Gründung und dem Aufbau des Vereins zur Hilfe suchtmittelabhängiger Frauen e. V. beteiligt, deren Gesamtleitung und Geschäftsführung sie von 1991 bis 1996 übernahm (vgl. http://ulrike-kreyssig.de/profil). Anne Kurth war im Department für Psychologie der FU Berlin im Bereich Frauenforschung als wissenschaftliche Mitarbeiterin beschäftigt (Kurth 1994).

24 Kreyssig, Ulrike und Anne Kurth (1981). Die weibliche Sucht Emanzipation. *Sozialmagazin* [weitere Angaben unbekannt] in FFBIZ/ARep400, 1970–1984.

25 Plogstedt, Sybille 1988. Frauen und Gesundheit: Sucht und Emanzipation. In: Informationsdienst des Deutschen Frauenrates Bundesvereinigung der Deutschen Frauenverbände und Frauengruppen gemischter Verbände e. V., 37 (7–8), S. 3–5, hier S. 4, in FFBIZ, ARep400, BRD 14a (1979–2001).

26 Valverde zeigt für die USA auf, dass insbesondere Selbsthilfeansätze wie der der Anonymen Alkoholiker in den USA auch von Ärzt:innen am häufigsten empfohlen wurden, da sie diese für am effektivsten hielten.
